# Metabolomics and Transcriptomics Revealed a Comprehensive Understanding of the Biochemical and Genetic Mechanisms Underlying the Color Variations in Chrysanthemums

**DOI:** 10.3390/metabo13060742

**Published:** 2023-06-10

**Authors:** Di Wu, Fengchao Zhuang, Jiarui Wang, Ruiqi Gao, Qiunan Zhang, Xiao Wang, Guochao Zhang, Minghui Fang, Yang Zhang, Yuhua Li, Le Guan, Yanqiang Gao

**Affiliations:** 1 Key Laboratory of Saline-Alkali Vegetation Ecology Restoration, Ministry of Education, Northeast Forestry University, Harbin 150040, China; wudi000809@163.com (D.W.);; 2 College of Life Science, Northeast Forestry University, Harbin 150040, China

**Keywords:** chrysanthemum, flower color, widely targeted metabolome, transcriptome, anthocyanins, biomarker

## Abstract

Flower color is an important characteristic of ornamental plants and is determined by various chemical components, including anthocyanin. In the present study, combined metabolomics and transcriptomics analysis was used to explore color variations in the chrysanthemums of three cultivars, of which the color of JIN is yellow, FEN is pink, and ZSH is red. A total of 29 different metabolites, including nine anthocyanins, were identified in common in the three cultivars. Compared with the light-colored cultivars, all of the nine anthocyanin contents were found to be up-regulated in the dark-colored ones. The different contents of pelargonidin, cyanidin, and their derivates were found to be the main reason for color variations. Transcriptomic analysis showed that the color difference was closely related to anthocyanin biosynthesis. The expression level of anthocyanin structural genes, including *DFR*, *ANS*, *3GT*, *3MaT1*, and *3MaT2*, was in accordance with the flower color depth. This finding suggests that anthocyanins may be a key factor in color variations among the studied cultivars. On this basis, two special metabolites were selected as biomarkers to assist in chrysanthemum breeding for color selection.

## 1. Introduction

Chrysanthemums (*Chrysanthemum morifolium*) originated in China and were first cultivated in China as herbs around the 15th century BC [1]. Afterward, they were introduced in Japan (8th century AD), Europe (the 17th Century), and the United States (the 18th Century) [2]. The plants were selected and improved in each country to give rise to thousands of cultivars with flowers of various forms and colors. Through long-term artificial selection, chrysanthemums have more than 30,000 cultivars, and approximately 3000 of which are grown in China [3]. Today, the chrysanthemum has been widely cultivated and become one of the most important floricultural crops worldwide because of its great ornamental and economic value. In China, the chrysanthemum is one of the four prominent cut flowers. The cultivation area, production, and sales have always been at the forefront of the global cut flower industry [4].

Chrysanthemums vary considerably in terms of flower color, morphology, scents, and so on. The flower color is one of the most important ornamental traits determining its economic value. The color of the disc floret is generally yellow or green, whereas petals of the ray floret (ray petals) exhibit a diverse range of colors, mainly including white, green, pink, yellow, red, orange, brown, and other color groups [5]. These petal colors are formed by the accumulation of a single pigment, a combination of multiple pigments, such as anthocyanins, carotenoids, and chlorophylls, or the absence of these pigments [6,7,8]. The color of yellow chrysanthemum flowers consisted of flavones and carotenoids, that of white flowers consisted of flavones, that of red and purple flowers consisted of anthocyanins and other flavonoids, and that of orange flowers consisted of carotenoids, anthocyanins, and other flavonoids. Park et al. [9] reported that chrysanthemum flowers with high amounts of anthocyanins displayed a wide range of red and purple colors. Those with higher carotenoid contents presented yellow and green colors. Several eye-catching examples include β-carotene from carrots and sweet potatoes, lycopene from tomatoes and watermelon, capsanthin and capsorubin from red peppers, and lutein from marigold flowers. However, the relationship between the secondary metabolites of chrysanthemum flowers and their color variations remains unclear.

Anthocyanins are a group of widely distributed water-soluble compounds that make a significant contribution to the color development of many ornamental plants [10,11]. Anthocyanins strengthen plants’ capacity to adapt to environmental stresses such as pathogen infection, UV radiation, drought, and low temperatures, in addition to enhancing their aesthetic value [12,13,14]. Anthocyanins have a basic flavonoid parent nucleus. There are six major forms of anthocyanidins in the plant, depending on the B-ring substituents: delphinidin, petunidin, cyanidin, malvidin, pelargonidin, and peonidin [15,16]. Anthocyanin anabolic pathways have been extensively studied [17,18]. Naringenin chalcone was generated by 1 p-coumaroyl-CoA and 3 malonyl-CoA under the catalyzing of Chalcone synthase (CHS) [19]. Chalcone isomerase (CHI) quickly converts it into naringenin. Naringenin is then transformed to dihydrokaempferol by F3H, as well as via the subsequent action of dihydrokaempferol to dihydroquercetin in F3′H and dihydromyricetin in F3′5′H [20]. Later, a variety of anthocyanins are generated in dihydroflavonol 4-reductase (DFR), anthocyanin synthase (ANS), O-methyltransferases (OMT), and UDP glycosyltransferase (UGT), as well as other enzymes [21].The anthocyanin biosynthesis pathway is relatively conserved in plants and is regulated by various transcription factors (TFs). A transcription complex formed by MYB, bHLH, and WD40 acts as a switch to regulate anthocyanin synthesis. Among them, numerous MYB family members are reported to transcriptionally activate anthocyanin biosynthetic genes or repress anthocyanin accumulation in plant food such as apples [22], peppers [23], and sweet potatoes [24].

Herein, metabolomic and transcriptomic analysis were performed in this study. Three chrysanthemum cultivars’ differently accumulated metabolites and differentially expressed genes were identified. Metabolomic analysis found that the content of pelargonidin, cyanidin, and their derivates significantly increased in the dark-colored cultivars compared to the light-colored ones. Transcriptome data and pathway analysis revealed that anthocyanin biosynthesis pathways gradually increased with the change in flower color. An in-depth investigation of the variations in anthocyanin derivatives and the chromogenic mechanism of two heterochromatic flowers of the same plant, as well as an analysis of the variations in metabolomics and transcriptomics of plants with different colors, managed to give new insights for the breeding of chrysanthemums in the future.

## 2. Materials and Method

### 2.1. Materials

All of the chrysanthemum cultivars in this study (*Chrysanthemum morifolium*, a ground-cover chrysanthemum cultivar) were cultivated and planted in the Flower Research Institute of the College of Life Sciences, Northeast Forestry University, China. The twelve cultivars were “Yingxue” (YX), “Xuehaibaifan” (XHBF), “Jinshuangdie” (JIN), “Jinqiu” (JQ), “Fenshaqun” (FSQ), “Wanxia” (WX), “Fenshuangdie” (FSD), “Fenselangman” (FSLM), “Zirong” (ZR), “Zitao” (ZT), “Yingshanhong” (YSH), and “Zhushahong” (ZSH). All plants were cultivated in a greenhouse at day/night temperatures of 24 °C/18 °C and an 8/16 h light/dark photoperiod. They were then transplanted into outdoor fields in May to grow naturally and were observed until October. According to the development of the inflorescence, the perianth is divided into 6 developmental stages (S1–S6): S1, bracts are not open and are tightly wrapped; S2, the flower bud is open but ray florets are not visible; S3, the flower bud is open and ray florets are visible; S4, the flower bud is open and the tips of florets are visible; S5, the outer florets are elongated and are obliquely oriented; and S6, the outer florets are horizontally oriented. Petals were collected from 20 flowers in the S4 stage, and 3.0 g petals were taken from each sample for metabolomic and transcriptomic detection.

### 2.2. Equipment

The chrysanthemum petal samples were freeze-dried in a vacuum and then ground to powder form using a grinder (30 Hz, 1.5 min). A total of 100 mg of the powder was then weighed and dissolved in 0.6 mL of 70% methanol extract. The dissolved samples were placed in a 4 °C refrigerator overnight and vortexed six times to improve the extraction rate. Samples were then centrifuged at 10,000× *g* for 10 min, and the supernatant was absorbed and filtered using a microporous filter membrane. The supernatant of each sample was filtered using microporous filtration and analyzed using high-performance liquid chromatography–tandem mass spectrometry (HPLC-MS/MS) using an Applied Biosystems 4500 Qtrap mass spectrometer equipped with the Shim-pack UFLC Shimadzu CBM30A system (Agilent Technologies, Santa Clara, CA, USA). Analytes were separated using a Waters Acquity UPLC HSS T3 C18 column (Waters, Milford, MA, USA) (2.1 mm × 100 mm, 2.8 μm) at 40 °C. Double-distilled water containing 0.04% acetic acid (*v*/*v*; solvent A) and acetonitrile containing 0.04% acetic acid (*v*/*v*; solvent B) were used as the mobile phase at a flow rate of 0.35 mL min^−1^. The gradient program was conducted as follows: 0 min, 5% B; 0–10 min, 95% B; and 11–14 min, 5% B. The injection volume was 4 μL. The mass spectrometer was operated in the negative ion mode, and the electrospray ionization (ESI) source parameters were as follows: drying gas temperature, 550 °C; mass spectrum voltage, 5500 V; curtain gas (CUR), 30 psi; and collision-activated dissociation (CAD), high. Each ion was scanned and detected based on the decluttering potential (DP) and collision energy (CE). The analysis was performed with three biological replicates.

### 2.3. Transcriptome Assays

Total RNA extraction, quality inspection, DNase I treatment, and rRNA removal (using the Ribo-Zero kit; Epicentre, Madison, WI, USA) were performed as described previously [25]. Fragment buffers were added to each sample and the RNA was cut into fragments of 200–500 bp. The first-strand cDNA was synthesized from the RNA fragment using random hammerer primers. Then, using the first strand of cDNA as a template, the second strand of cDNA was synthesized with dUTP instead of dTTP. AMPure XP beads (Beckman Coulter, Brea, CA, USA) were used for fragment size selection and the selected cDNA fragments were amplified via polymerase chain reaction (PCR). Finally, the cDNA library was sequenced using the Illumina HiSeq X Ten system (Illumina, San Diego, CA, USA) from Chengdu Biobaseline Technology Co., LTD. (Chengdu, China).

### 2.4. PCA and OPLS-DA

The original data were compressed into n principal components to describe the features of the original data set. PC1 represents the most obvious features that can be described in the multidimensional data matrix, PC2 represents the most significant features that can be described in the matrix except PC1, and so on. PCA uses the built-in statistical prcomp function of R software (www.r-project.org) and sets prcomp function parameter scale = TRUE, which means normalizing the data via unit variance scaling (UV).

Orthogonal partial least squares discriminant analysis (OPLS-DA) combines orthogonal signal correction (OSC) and PLS-DA methods, which can decompose the information of the X matrix into two types of information related and unrelated to Y, and screen the differential variables by removing the unrelated differences. After log2 conversion of the original data, OPLS-DA carries out centralized processing (mean focus), and uses the MetaboAnalystR package OPLSR. The anal function in R software for analysis analyzes metabolome data according to the OPLS-DA model, and draws the score chart of each group. The differences between groups were further demonstrated [26]. The prediction parameters of the evaluation model included R^2^X, R^2^Y, and Q^2^, in which R^2^X and R^2^Y, respectively, represent the interpretation rate of the built model to the X and Y matrix, and Q^2^ represents the prediction ability of the model. The closer the three indexes are to 1, the more stable and reliable the model is. When Q2 > 0.5, it can be considered as an effective model, and when Q2 > 0.9, it is an excellent model. The arrangement verification of OPLS-DA was carried out (n = 200, that is, 200 arrangement experiments were conducted). In model verification, the horizontal line corresponded to R^2^Y and Q^2^ of the original model, and the red and blue dots represented the R^2^Y′ and Q^2^′ of the model after Y replacement. If R^2^Y′ and Q^2^′ are both smaller than R^2^Y and Q^2^ of the original model, that is, the corresponding points do not exceed the corresponding lines, then the model is meaningful.

### 2.5. Statistics Analysis

The metabolites with a fold change > 2 and a fold change ≤ 0.5 were selected. If the difference in metabolites between the control group and the experimental group was more than 2 times or less than 0.5 times, the difference was considered to be significant. For samples with biological duplication, DESeq2 [27,28] was suitable for differential expression analysis between the sample groups to obtain differential expression gene sets between two biological conditions. DESeq2 requires unstandardized read counting data of input genes, rather than standardized RPKM, FPKM, and other data. The count of gene reads was achieved using featureCounts [29]. After difference analysis, it is also required that the Benjamini–Hochberg method be used to correct multiple hypothesis testing probabilities (P value) and obtain the false discovery rate (FDR). There were differences in the gene screening conditions for|log2Fold Change| ≥ 1 and FDR < 0.05.

### 2.6. KEGG and GO

The Kyoto Encyclopedia of Genes and Genomes (KEGG, https://www.kegg.jp/) is an integration of genome, biological pathways, disease, medicines, and chemical information, and is a comprehensive database [30]. The KEGG database was used to annotate differential accumulation metabolites and differential genes. Enrichment analysis of differentially expressed genes was performed using the cluster Profiler R package.

## 3. Results

### 3.1. UPLC-ESI-MS/MS-Based Quantitative Metabolomic Analysis

**Figure 1:**
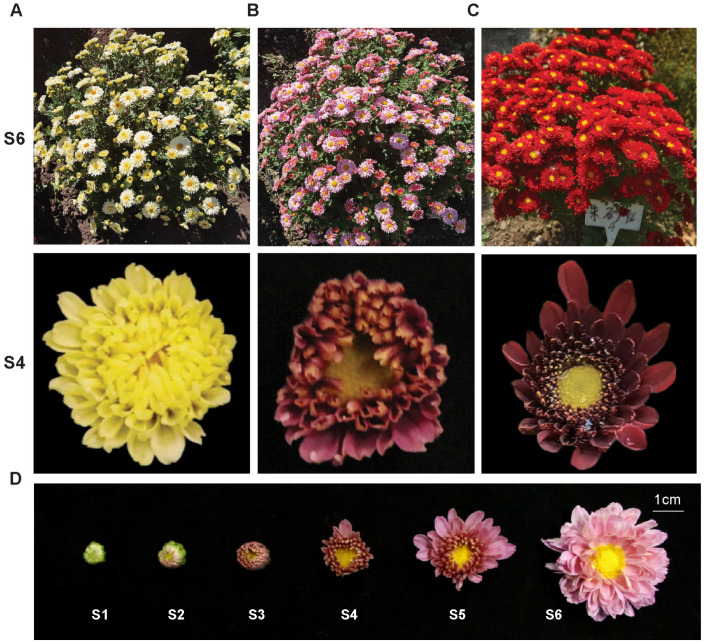
Phenotypes of different chrysanthemums. (**A**–**C**) Phenotypes of JIN, FEN, and ZSH, respectively. The upper of S6 is the phenotype of the whole plant in stage 6, the lower of S4 is the specific flower phenotype in stage 4. (**D**) Six flower developmental stages, defined by the extent to which the petals unfold.

To understand how metabolites vary corresponding to flower colors, we performed a widely targeted metabolite analysis of three diverse samples to obtain a comprehensive metabolic profile based on ultra-performance liquid chromatography–electro spray ionization–tandem mass spectrometry (UPLC-ESI-MS/MS). Ray petals of JIN, FEN, and ZSH, whose colors were yellow, pink, and red, respectively, were collected in the S4 stage. Among the three cultivars, JIN and FEN share a similar genetic background [31] (Figure 1).

**Figure 2:**
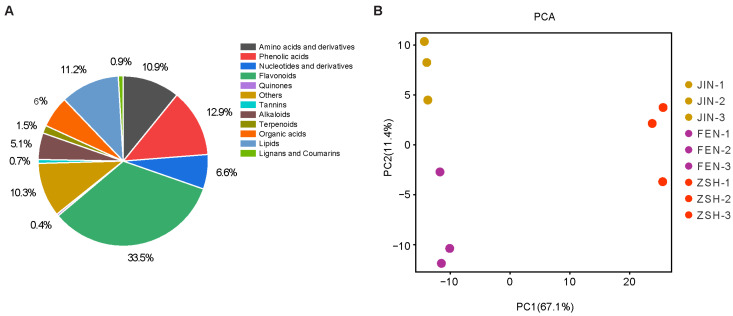
Metabolomic analysis of chrysanthemums. (**A**) Classification of metabolites detected. (**B**) The principal component analysis of different chrysanthemum cultivars; PC1 and PC2 are the first and second principal components.

A total of 732 metabolites were determined using the UPLC-MS/MS system, including alkaloids (5.1%), amino acids and derivatives (10.9%), flavonoids (33.5%), lignans and coumarins (0.9%), lipids (11.2%), nucleotides and derivatives (6.6%), organic acids (6.0%), phenolic acids (13.93%), quinones (0.4%), tannins (0.9%), terpenoids (1.5%), and others (10.3%) (Figure 2A). The color formation of flavonoids is related to the existence of a cross-conjugated system, as well as the type, number, and position of substituent groups [32]. Anthocyanins, as a kind of flavonoids, have a great influence on the color of plants. In this study, a total of 176 flavonoids were detected, of which 13 were anthocyanins. The anthocyanins detected can be divided into four categories, which are pelargonidin, cyanidin, peonidin, and delphinidin, and their derivatives.

Principal component analysis (PCA) is a multidimensional statistical analysis method for unsupervised pattern recognition. PCA was performed on the three groups of samples to assess metabolic differences. Subjecting the metabolite data to PCA separated the three cultivars regarding metabolite composition, which demonstrated that significant differences existed among the metabolite levels within these cultivars, especially regarding ZSH and the other two cultivars. The first principal component (PC1) contributed to 67.1% of the variation, whereas PC2 was associated with 11.4% of the variation (Figure 2B). The first component (PC1) separated ZSH and FEN, while the second component (PC2) separated JIN and FEN. This may be due to the fact that JIN and FEN are derived from the same genetic resource [31].

Orthogonal partial least squares discriminant analysis (OPLS-DA) can decompose the information of the X matrix into two types of information related and unrelated to Y, and screen the differential variables by removing the unrelated differences. The OPLS-DA model was used to compare the samples in pairs to observe the differences in metabolites between the samples. The three groups of samples were compared in pairs and scored using the OPLS-DA model. The results showed that the inter-group difference scores of JIN vs. FEN, JIN vs. ZSH, and FEN vs. ZSH were 41.4%, 75.6%, and 72.9%, respectively. The T-score of JIN and FEN from the same genetic line was significantly lower than that of the others, which was consistent with the results of PCA. To verify this result, two hundred alignment experiments were then conducted. The facts proved that our conclusion was credible (Figure S1).

### 3.2. Analysis of Differentially Accumulated Metabolites (DAMs)

**Figure 3:**
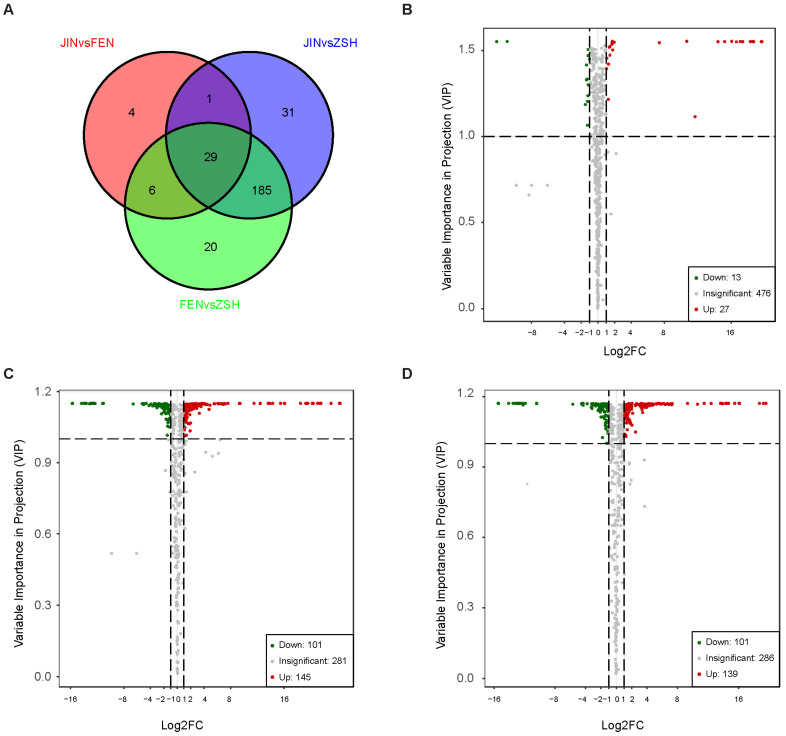
Analysis of the DAMs in chrysanthemums. (**A**) Venn diagram shows the DAMs shared by JIN vs. FEN, JIN vs. ZSH, and FEN vs. ZSH. (**B**) Volcano plot of DAMs for JIN vs. FEN, JIN vs. ZSH (**C**), and FEN vs. ZSH (**D**).

In the samples of JIN, FEN, and ZSH, 498, 509, and 507 metabolites were detected, respectively, and among which, 473 metabolites were shared by the three groups. We found a total of 29 different metabolites in common in the three groups of samples, including nine anthocyanins. Interestingly, all nine anthocyanins were up-regulated (Figure 3A). To further explore the metabolite accumulation pattern, we screened them based on PCA and OPLS-DA results, as well as fold change and VIP (variable importance in projection) data. In the JIN vs. FEN group, 40 differential metabolites were found, of which 27 metabolites were up-regulated and 13 metabolites were down-regulated (Figure 3B). For JIN vs. ZSH, 246 differential metabolites were found, of which 145 metabolites were up-regulated and 101 metabolites were down-regulated (Figure 3C). For FEN vs. ZSH, 139 metabolites were up-regulated and 101 were down-regulated (Figure 3D).

**Figure 4:**
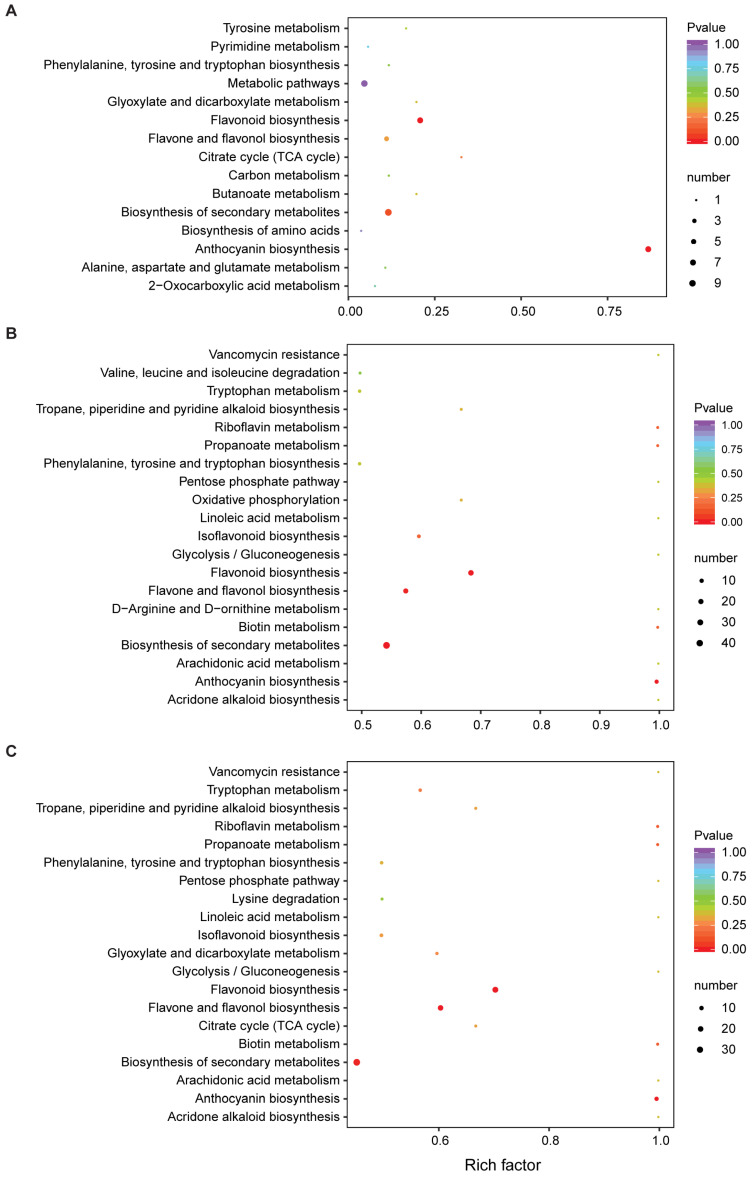
The KEGG pathways of DAMs in different comparison groups. (**A**) JIN vs. FEN, (**B**) JIN vs. ZSH, (**C**) FEN vs. ZSH.

As a major public pathway database, the KEGG database is helpful for researchers to study genes, expression information, and metabolite content as a whole network, and it is a powerful tool for metabolism analysis and metabolic network research in vivo. In this study, the differential metabolites of each control group were annotated and enriched, and divided into different KEGG pathways. KEGG enrichment analysis showed that the DAMs in the JIN vs. FEN group were significantly enriched in the anthocyanin biosynthesis pathway, followed by the flavonoid biosynthesis and secondary metabolite biosynthesis pathway (Figure 4A). The DAMs for the JIN vs. ZSH and FEN vs. ZSH groups were also significantly enriched in the anthocyanin biosynthesis pathway, besides the flavone and flavonol biosynthesis pathway, riboflavin metabolism, and biotin metabolism (Figure 4B,C). This indicates that the DAMs in the anthocyanin biosynthesis pathway may be the key for the color differences among the three groups of chrysanthemum samples, especially in JIN and FEN.

**Figure 5:**
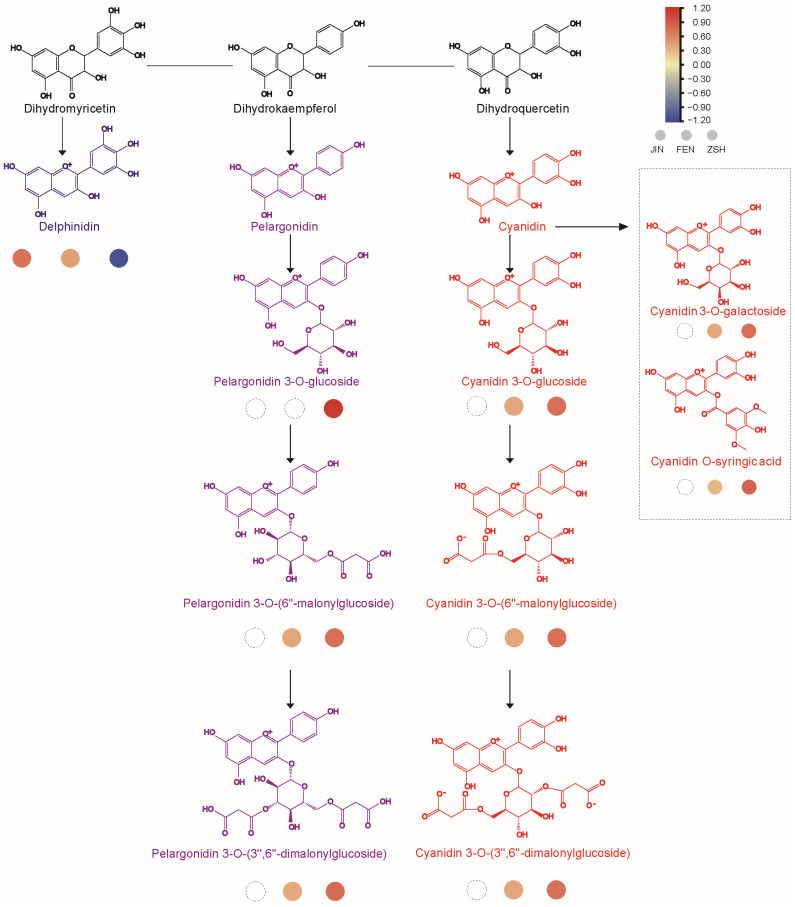
Metabolite accumulation model of anthocyanins and their derivatives in the three cultivars. The three circles represent JIN, FEN, and ZSH from left to right, and the color of the circles from blue to red indicates the accumulation of metabolites from low to high. The dashed circle indicates that the substance was not detected.

To further explore the relationship between flower color and anthocyanins, we analyzed the content of anthocyanins in three samples. The results showed that 13 anthocyanins were detected, and of which, 9 were significant DAMs, including pelargonidin 3-O-(6′′-malonylglucoside), pelargonidin 3-O-(3′′,6′′-dimalonylglucoside), cyanidin 3-O-galactoside, cyanidin 3-O-glucoside, cyanidin 3-O-(6′′-malonylglucoside), cyanidin O-syringic acid, cyanidin 3-O-(3′′,6′′-dimalonylglucoside), cyanidin 3-O-(3′′′′,6′′′′-diacetylhexoside)-O-glyceric acid, and Cyanidin 3-O-(6′′-malonylglucoside)-5-Glucoside (Figure 5). Only peonidin, delphinidin, and rosinidin O-hexoside were detected in JIN. Interestingly, we detected 12 anthocyanins in FEN except for pelargonidin 3-O-glucoside. However, the acylation products of pelargonidin 3-O-glucoside such as pelargonidin 3-O-(6′′-malonyl glucoside) and pelargonidin 3-O-(3′′,6′′-dimalonylglucoside) were high in content. We determined that pelargonidin 3-O-glucoside is rapidly acylated to downstream substances after synthesis. Different from FEN and JIN, 13 anthocyanins could be detected in ZSH. In particular, the glycosylation and acylation products of pelargonidin and cyanidin had high content. Previous studies have shown that pelargonidin 3-O-glucoside and cyanidin 3-O-glucoside and their modified products are important for the purplish-red or red color [33,34]. Although peonidin and delphinidin also had some effect on flower color, there was no significant difference between the two substances in the three cultivars. The results showed that the differences between FEN and JIN were mainly derived from pelargonidin and cyanidin and their derivatives in anthocyanins. The red color of ZSH was also due to the increased content of these metabolites (Table S1).

### 3.3. Analysis of Differentially Expressed Genes (DEGs)

In order to investigate the gene expression changes in JIN, FEN, and ZSH, RNA libraries were constructed using the Illumina platform and sequenced. After filtering the raw data and checking the sequencing error rate and GC content distribution, about 72.84 Gb of clean data was obtained from the nine cDNA libraries (three replicates per sample), ranging from 6.11 to 10.7 Gb per library, and the percentage of Q30 bases was 91% or above. Heatmap analysis of the samples based on FPKM values showed that all biological replicates showed similar expression patterns, indicating the high reliability of our sequencing data. These data demonstrate that the sequencing quality is sufficient for further analysis.

**Figure 6:**
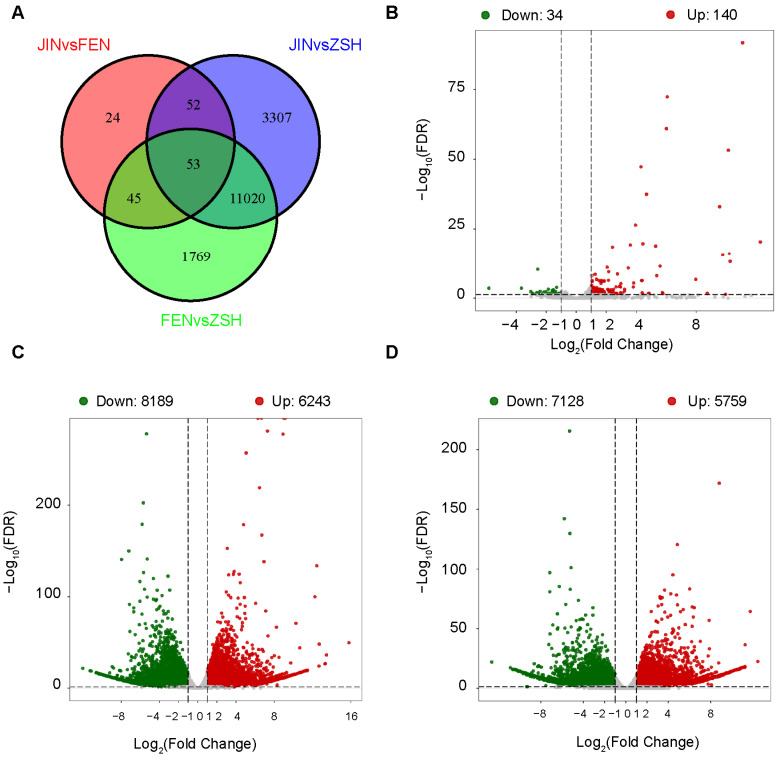
Analysis of the DEGs in chrysanthemums. (**A**) Venn diagram shows the DEGs shared by JIN vs. FEN, JIN vs. ZSH, and FEN vs. ZSH. (**B**) Volcano plot of DEGs for JIN vs. FEN, JIN vs. ZSH (**C**), and FEN vs. ZSH (**D**).

By analyzing the transcriptome data, 16,270 differentially expressed genes (DEGs) were detected in all of the samples. A Venn diagram showed that 53 DEGs were co-expressed in the three groups, with 41 genes up-regulated and 12 genes down-regulated (Figure 6A). JIN vs. FEN, JIN vs. ZSH, and FEN vs. ZSH had 174 DEGs (140 up-regulated genes, 34 down-regulated genes), 14,432 DEGs (6243 up-regulated genes, 8189 down-regulated genes), 12,887 DEGs (5759 up-regulated genes, 7218 down-regulated genes), and differential genes, respectively (Figure 6B–D). In common DEGs, we found the structural genes involved in anthocyanin synthesis, such as *CHS*, *F3H*, *DFR*, *ANS*, *3GT*, *3MaT1*, and *3MaT2*, and the transcription factor MYB6 to be associated with the anthocyanin biosynthesis pathway. These results indicate that the genes participating in the anthocyanins pathway have an effect on flower color.

**Figure 7:**
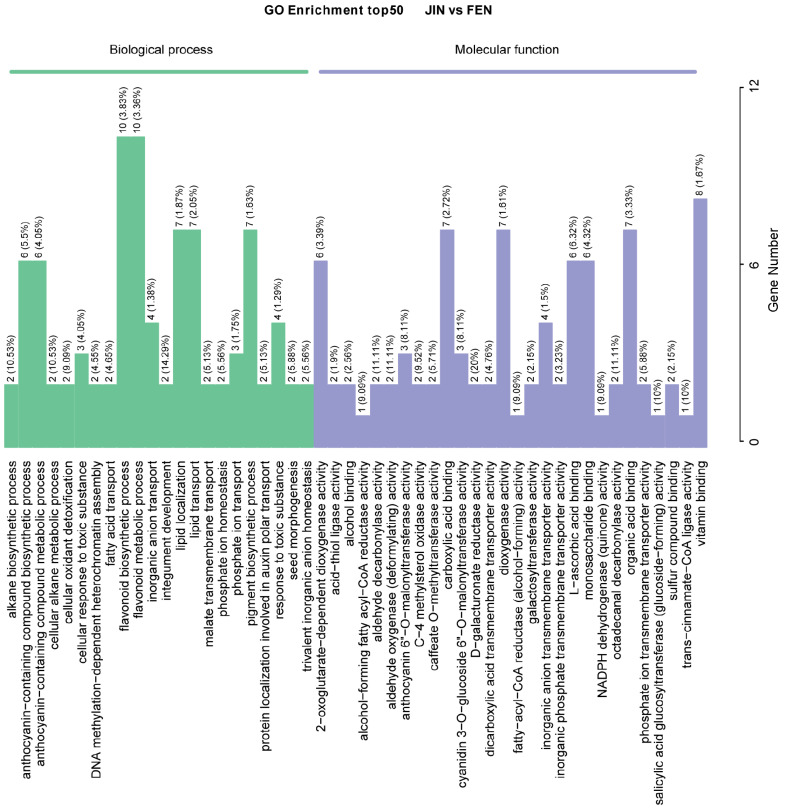
The GO enrichment of DEGs for JIN vs. FEN.

The results of GO enrichment analysis showed that the DEGs in the JIN vs. FEN group were significantly enriched in two modules of biological process and molecular function (Figure 7). In the biological process modules, there was the anthocyanin-containing compound biosynthetic process and the anthocyanin-containing compound metabolic process; the flavonoid biosynthetic process and flavonoid metabolic process showed significant enrichment. In the molecular function module, anthocyanin 6′′-O-malonyltransfer-ase activity, cyanidin 3-O-glucoside 6′′-O-malonyltransferase activity, D-galacturonate reductase activity, and galactosyltransferase activity were significantly enriched, which is closely related to the anthocyanin synthesis pathway (Figure 7). JIN is genetically similar to FEN, while ZSH is not. GO enrichment analysis of DEGs in both groups indicates the apparent enrichment of the flavonoid metabolic process. In addition, cinnamate β−D−glucosyltransferase activity and flavone7−O−β−glucosyltransferase activity were also enriched for FEN vs. ZSH (Figures S2 and S3).

**Figure 8:**
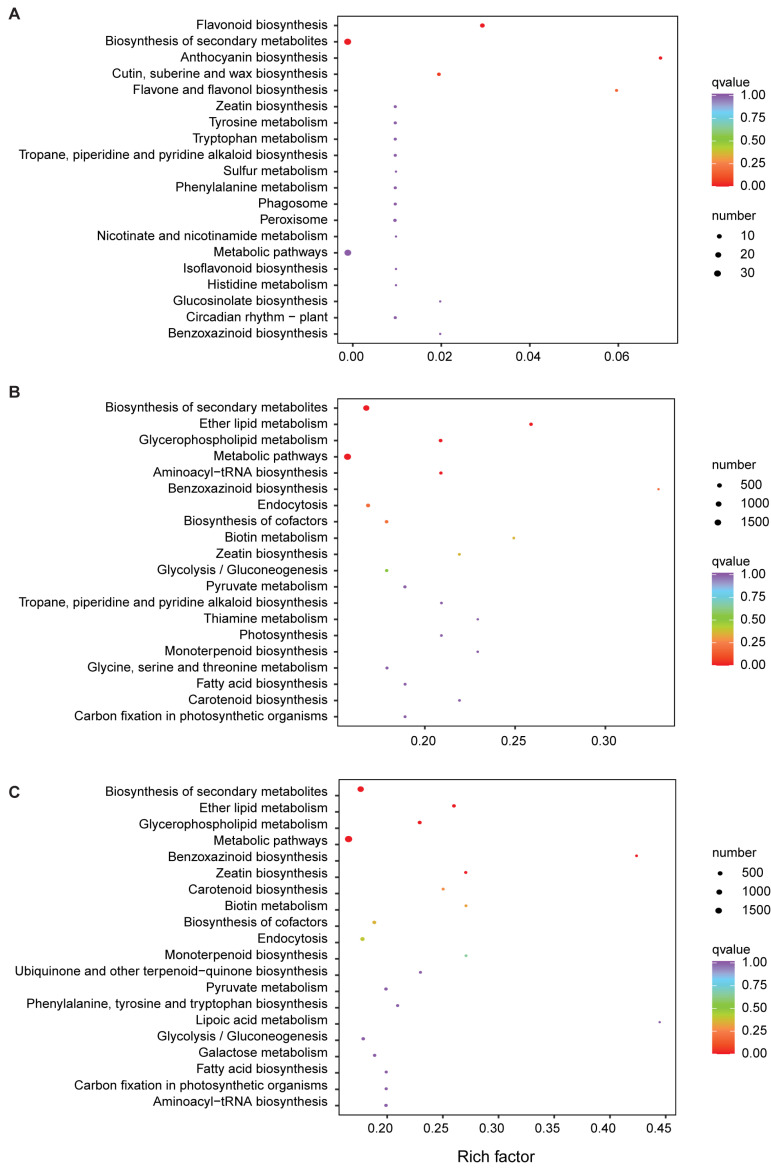
The KEGG pathways of DEGs in different comparison groups. (**A**) JIN vs. FEN, (**B**) JIN vs. ZSH, (**C**) FEN vs. ZSH.

In order to further explore the causes of color differences, we performed KEGG analysis. Not surprisingly, JIN vs. FEN’s DEGs were significantly enriched for flavonoid biosynthesis, anthocyanin biosynthesis, and flavone and flavanol biosynthesis (Figure 8A). It was further confirmed that anthocyanin was the important factor affecting the difference between them. For JIN vs. ZSH, apart from the metabolic pathways and biosynthesis of secondary metabolites, ten DEGs were enriched in the anthocyanin biosynthesis pathway and eight were enriched for flavone and flavanol biosynthesis (Figure 8B). In addition, FEN vs. ZSH also had 10 DEG annotations in the anthocyanin biosynthesis pathway (Figure 8C).

### 3.4. Anthocyanin Biosynthesis Pathway

**Figure 9:**
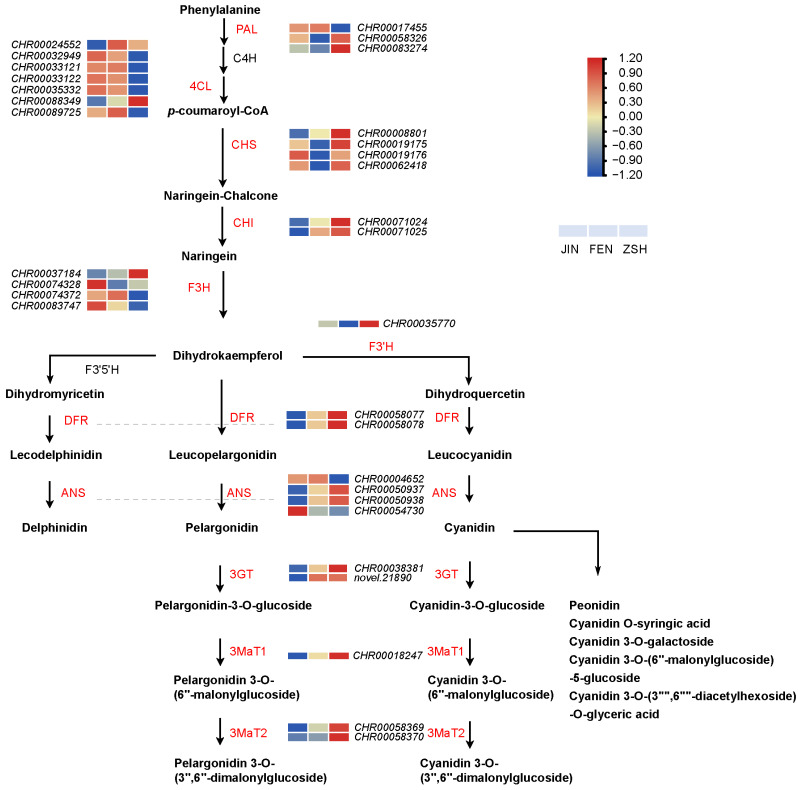
Expression levels of structural genes in anthocyanin biosynthesis pathways in chrysanthemums. All cells represent the log_2_FPKM of the genes. FPKM > 10 is considered to be significant. PAL, phenylalanine ammonia lyase; C4H, cinnamate 4-hydroxylase; 4CL, 4-coumarate: CoA ligase; CHS, chalcone synthase; CHI, chalcone isomerase; F3H, flavanone 3-hydroxylase; F3′H, flavonoid 3′-hydroxylase; F3′5′H, flavonoid 3′,5′-hydroxylase; DFR, dihydroflavonol 4-reductase; ANS, anthocyanidin synthase; 3GT, UDP-glucose: anthocyanidin 3-glucosyltransferase; 3MaT1, anthocyanin 3-O-glucoside-6′′-O-malonyltransferase; 3MaT2, anthocyanidin 3-O-glucoside-3′′,6′′-O-dimalonyltransferase.

We mapped the anthocyanin biosynthesis pathways, including heatmaps of the detected structural gene expression level (Figure 9). The genes encoding PAL (phenylalanine ammonia lyase, *CHR00017455*, *CHR00058326*, *CHR00083274*) and 4CL (4-coumarate-CoA ligase, *CHR00033121*, *CHR00033122*, *CHR00035332*, *CHR00088349*, *CHR00089725*) were detected as important genes of the phenylpropane metabolic pathway, and their expression levels were not correlated with flower color.

We found that the gene encoding CHS (CHR0008801) was expressed in FEN and ZSH but not in JIN. Its expression level in ZSH was about 50 times that of FEN, which may be an important structural gene affecting flower color change. Although other CHS coding genes (*CHR00019175*, *CHR00019176*, *CHR00062418*) were detected, there was no significant difference in their expression levels. The expression level of CHI (*CHR00071024*, *CHR00071025*) and F3H (flavanone 3-hydroxylase, *CHR00037184*) increased with the change in flower color, but *F3H* (*CHR00074328*, *CHR00074372*, *CHR00083747*) did not. F3′5′H (flavonoid 3′5′-hydroxylase) and F3′H (flavonoid 3′-hydroxylas), as the key branch points of the anthocyanin biosynthesis pathway, catalyzed the synthesis of dihydrokaempferol to form dihydromyricetin and dihydroquercetin, respectively. Interestingly, only the structural gene of F3′H (*CHR00035770)* was detected.

The genes encoding DFR (*CHR00058077*, *CHR00058078*) and ANS (*CHR00050937*, *CHR00050938*) are key genes in the downstream of the anthocyanin biosynthesis pathway. 3GT (UDP-glucose: anthocyanidin 3-glucosyltransferase, *CHR00038381, novel.21890*), *3MaT1* (anthocyanin 3-O-glucoside-6′′-O-malonyltransferase, *CHR00018247*), and *3MaT2* (anthocyanidin 3-O-glucoside-3′′,6′′-O-dimalonyltransferase, *CHR00058369*, *CHR00058370*) were the key enzymes for the glycosylation and acylation of pelargonidin and cyanidin to form stable anthocyanins. As expected, these genes were not expressed in JIN, and their expressions in ZSH were much higher than those in FEN. The difference in the expression of these genes may be an important reason for the change in flower color.

### 3.5. Verification of Metabolite Accumulation Patterns with Nine Different Color Cultivars and Identification of Two Metabolites as Potential Biomarkers

Metabolomic analysis showed that pelargonidin and cyanidin and their derivatives were not accumulated in the yellow chrysanthemum cultivars but were highly accumulated in the red ones. This was also supported by the difference in the expression of structural genes in the anthocyanin biosynthetic pathway. To further verify these results, we selected another nine cultivars with yellow, pink, and red colors, respectively, to analyze their anthocyanin composition. The result showed that the accumulation patterns of these metabolites in the three groups of chrysanthemums with different petal colors are highly consistent with the above results.

**Figure 10:**
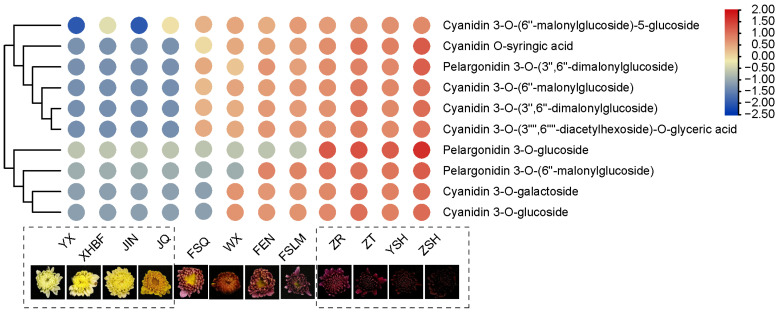
The heatmap of anthocyanins in twelve chrysanthemum cultivars.

In the yellow chrysanthemum cultivar group, the results showed that pelargonidin and cyanidin and their derivatives were not detected (YX, XHBF, JIN, and JQ). Interestingly, pelargonidin 3-O-glucoside was not detected in all of the pink cultivar groups (FSQ, WX, FEN, and FSLM), whereas its malonylated form pelargonidin 3-O-(6′′-malonyglucoside) existed both in red and pink cultivars, except in FSQ and WX. However, pelargonidin 3-O-(3′′,6′′-dimalonylglucoside) could be detected in all pink groups. The possible reason was due to the rapid secondary malonylation of pelargonidin 3-O-(6′′-malonyglucoside) in FSQ and WX. The reason why pelargonidin and their derivatives could not be clustered together was due to different accumulation patterns in the pink group. Cyanidin 3-O-glucoside was also not detected in the FSQ, but its acylation forms could be detected; the content was lower than that of the other three cultivars. This may be the reason why the phenotype of FSQ was still partially yellow. As expected, pelargonidin and cyanidin and their derivatives could be detected in the red group (ZR, ZT, YSH, and ZSH). If the contents were higher, the color would be more red (Figure 10).

**Figure 11:**
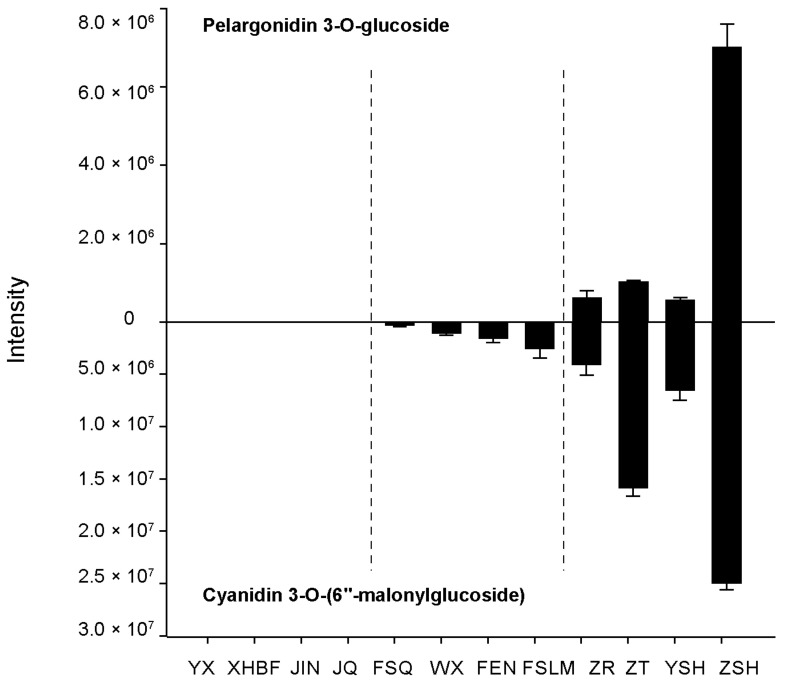
Intensity of pelargonidin 3-O-glucoside and cyanidin 3-O-(6′′-malonylglucoside) in twelve cultivars. Error bars are SDs of three biological replicates.

We further attempted to differentiate the color types (yellow, pink, and red) according the accumulation pattern of the following metabolites. Cyanidin 3-O-(6′′-malonylglucoside) could be detected in both the pink and red groups, but pelargonidin 3-O-glucoside was only present in the red groups. The yellow group did not have these two metabolites (Figure 11). Based on these findings, two potential biomarkers could be identified to determine the color of chrysanthemums. LC-MS or UPLC can be used to detect chrysanthemums. When pelargonidin 3-O-glucoside and cyanidin 3-O-(6′′-malonylglucoside) were detected at the same time, the flower color tended to be red, whereas when only pelargonidin 3-O-glucoside was detected, the color was likely to be pink. If neither were present, then there was a chance that the color would be yellow.

## 4. Discussion

As one of the most popular flower crops in the world, the chrysanthemum ranks second in the cut flower trade [35]. In addition to its ornamental value, the chrysanthemum contains a large number of nutritional and bioactive ingredients, and is therefore widely used in food and drug research and development industries [36]. With the increasing market demand for chrysanthemum cultivars, it is crucial to breed new cultivars with novel appearance, stress resistance, and excellent quality [37]. Anthocyanins are the most common basic pigments and play a key role in flower color formation [12]. The different types and amounts of anthocyanins in plant tissues lead to the distinctive color of flowers. In addition, anthocyanins attract pollinators and animals to help spread pollen and seeds [38]. Metabolomic analysis is a post-genomic technology designed to provide a comprehensive profile of all metabolites (estimates at least 200,000) in a typical plant and represents an important branch of systems biology [39]. Via metabolomic analysis, many researchers have deeply studied the differences in flavonoid metabolites in crops, fruits, and flowers under different conditions to provide new insights into genetic breeding [40,41,42,43,44]. In this study, metabolomics technology was used to determine and analyze the metabolite content of different chrysanthemum cultivars. A total of 732 metabolites were detected. In sum, 40 DAMs were detected for JIN vs. FEN, but 246 and 240 DAMs were detected for JIN vs. ZSH and FEN vs. ZSH, respectively. This may be because JIN and FEN are two chrysanthemum cultivars from the same genetic line, while ZSH, as the significant red cultivar, differs from them. KEGG enrichment analysis showed that the anthocyanin biosynthesis pathways were significantly enriched in all three groups. A total of 11 anthocyanins were detected in the three groups. We found that cyanidin and pelargonidin and their derivatives were not expressed in JIN, except for delphinidin and peonidin. On the contrary, FEN and ZSH detected these substances. Interestingly, pelargonidin 3-O-glucoside was not detected in FEN as it was rapidly converted to downstream substances after synthesis. The content of cyanidin and pelargonidin and their derivatives in ZSH was much higher than that in the other two cultivars. In general, with the increasing expression of cyanidin and pelargonidin, the red of the chrysanthemum increases gradually.

Transcriptomics is the study of the transcriptome, using high-throughput sequencing to specifically characterize and quantify the entire set of RNAs present in an organism’s organs, tissues, or cells [45]. Transcriptome analysis showed that 16,270 DEGs were detected. In the three groups, 41 genes were co-expressed and up-regulated, and it is worth noting that most of the up-regulated genes were related to enzymes of the anthocyanin biosynthesis pathway, or MYB transcription factors. GO enrichment analysis showed that the DEGs of JIN vs. FEN were significantly annotated in the anthocyanin-containing compound biosynthetic process, anthocyanin-containing compound metabolic process, flavonoid biosynthetic process, and flavonoid metabolic process. KEGG also showed that these DEGs were enriched in flavonoid biosynthesis and anthocyanin biosynthesis. This proves that the main reason for the color difference between JIN and FEN in the same genetic line is closely related to anthocyanins. Despite the great genetic difference between ZSH and the other two cultivars, the DEGs of JIN vs. ZSH and FEN vs. ZSH still annotated the entry of flavonoid metabolism in GO, and had gene enrichment to the anthocyanin biosynthesis pathway in KEGG.

We mapped the anthocyanin biosynthesis pathway and labeled the expression heatmap of synthase-related structural genes along the pathway. The upstream structural genes of the synthetic pathway did not change with different flower colors, and only a part of the structural genes showed such a behavior. Although the expression of some structural genes did not show a regular relationship with chrysanthemum flower color, they all had high expression levels. Only the structural gene of F3′H was detected, while F3′5′H, a competitive substance of F3′H, was not found. This may be related to the high expression of cyanidin and its derivatives. We found that the expressions of the DFR, ANS, 3GT, 3MaT1, and 3MaT2 structural genes in the downstream of the anthocyanin biosynthesis pathway changed regularly with the difference in flower color. When the color changed to red, the expression of these genes increased, which is consistent with the results of our metabolome.

Combined transcriptomic and metabolomic analysis proved that the color differences in JIN, FEN, and ZSH were mainly related to anthocyanin anabolism. Pelargonidin and cyanidin and their derivatives are important reasons for the formation of these differences. In addition, 12 chrysanthemum cultivars were divided into yellow, pink, and red groups and their anthocyanin metabolites were detected. Pelargonidin 3-O-glucoside and cyanidin 3-O-(6′′-malonylglucoside) were selected as biomarkers due to their specific accumulation in different groups. The standards of these two kinds of substances were easy to obtain and use, and the final flower color formation of chrysanthemum could be predicted via UPLC or LC-MS technology. These findings provide convenience for chrysanthemum horticulture breeding and genetic improvement.

## 5. Conclusions

In conclusion, this study revealed the differences in metabolomics and transcriptomics among the three chrysanthemum cultivars with varied flower colors, and the important effects of pelargonidin, cyanidin, and their derivatives on different flower color formation were described. We identified two potential biomarkers (cyanidin 3-O-(6′′-malonylglucoside) and pelargonidin 3-O-glucoside) to distinguish between different colored chrysanthemums in assisted breeding. These results provide new insights into color formation and could provide ideas for the genetic breeding of flowers with various colors.

## Data Availability

All of the data used in the article are provided in the supplementary file.

## Supplementary Materials

The following supporting information can be downloaded at https://www.mdpi.com/article/10.3390/metabo13060742/s1: Figure S1: Orthogonal partial least-squares analysis of different comparison groups; Figure S2: GO enrichment of DEGs for JIN vs. ZSH; Figure S3: GO enrichment of DEGs for FEN vs. ZSH; Table S1: The contents of anthocyanin and its derivatives in JIN, FEN, and ZSH; Table S2: FPKM of structural genes in the anthocyanin biosynthetic pathway.
