# Supplementary material for: Metabolomics and Transcriptomics Revealed a Comprehensive Understanding of the Biochemical and Genetic Mechanisms Underlying the Color Variations in Chrysanthemums
Source: Metabolites. 2023 Jun 10;13(6):742. doi: 10.3390/metabo13060742 (PMC10301146; doi:10.3390/metabo13060742)
Supplement: Supplementary file 1 [file metabolites-13-00742-s001.zip › Figure S1.pdf]

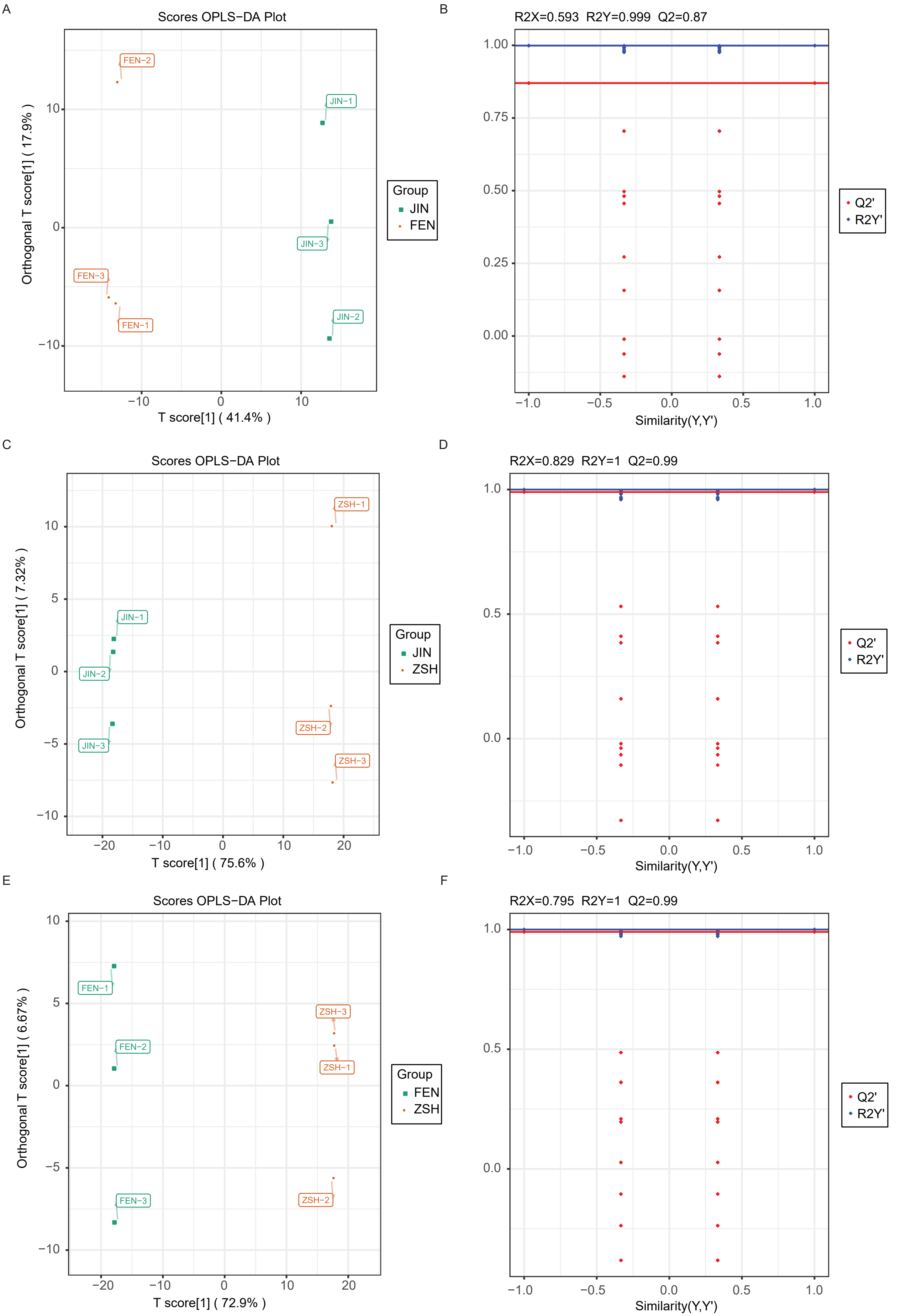

Figure S1. Orthogonal partial least-squares analysis of different comparison groups.(A,B) JIN vs FEN; (C,D) JIN vs ZSH; (E,F) FEN vs ZSH.
