# Supplementary material for: Metabolomics and Transcriptomics Revealed a Comprehensive Understanding of the Biochemical and Genetic Mechanisms Underlying the Color Variations in Chrysanthemums
Source: Metabolites. 2023 Jun 10;13(6):742. doi: 10.3390/metabo13060742 (PMC10301146; doi:10.3390/metabo13060742)
Supplement: Supplementary file 1 [file metabolites-13-00742-s001.zip › table S1.pdf]

Compounds	JIN	FEN	ZSH
Peonidin	9.81E+04	9.09E+04	2.19E+05
Delphinidin	1.18E+05	1.04E+05	5.07E+04
Pelargonidin 3-O-glucoside	Nd	Nd	7.00E+06
Pelargonidin 3-O-(6"-malonylglucoside)	Nd	3.86E+06	7.69E+07
Pelargonidin 3-O-(3",6"-dimalonylglucoside)	Nd	1.26E+06	2.69E+07
Cyanidin 3-O-galactoside	Nd	7.30E+06	1.97E+08
Cyanidin 3-O-glucoside	Nd	7.64E+06	1.86E+08
Cyanidin 3-O-(6"-Malonylglucoside)	Nd	1.55E+06	2.50E+07
Cyanidin O-syringic acid	Nd	1.97E+05	1.10E+07
Cyanidin 3-O-(3",6"-dimalonylglucoside)	Nd	9.12E+05	1.21E+07
Cyanidin 3-O-(3"",6""-diacetylhexoside)-O-glyceric acid	Nd	2.93E+06	2.50E+07
Cyanidin 3-O-(6"-Malonylglucoside)-5-Glucoside	Nd	3.37E+06	1.90E+07
Rosinidin O-hexoside	3.62E+04	2.88E+04	2.24E+04

Table S1. The contents of anthocyanin and its derivatives in JIN, FEN, and ZSH.”Nd”represent “Not detected”.
