# Supplementary figures and images for: Metabolomics and Transcriptomics Revealed a Comprehensive Understanding of the Biochemical and Genetic Mechanisms Underlying the Color Variations in Chrysanthemums
Source: Metabolites. 2023 Jun 10;13(6):742. doi: 10.3390/metabo13060742 (PMC10301146; doi:10.3390/metabo13060742)

GO Enrichment top50 JIN vs ZSH

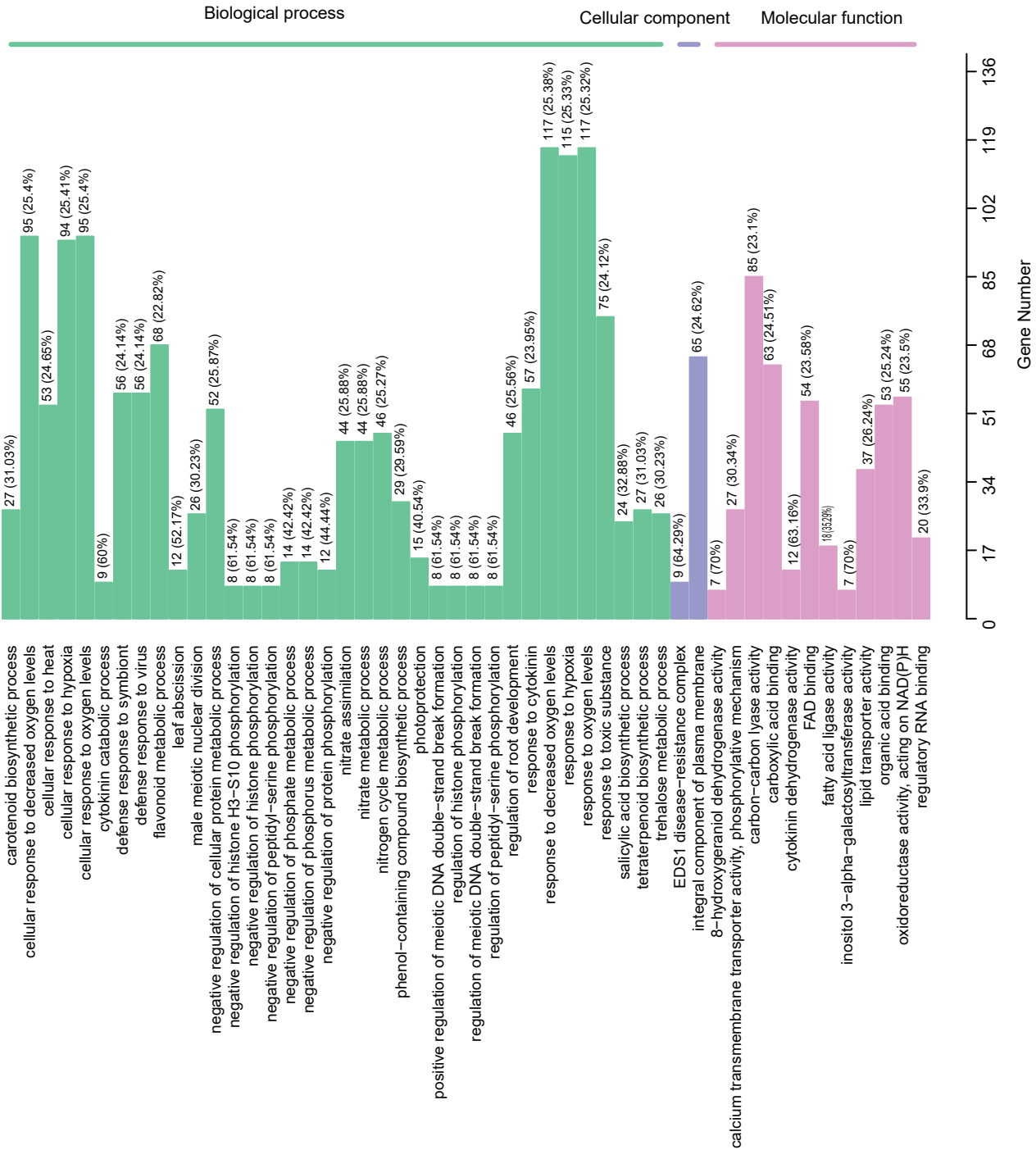

Figure S2. The GO enrichment of DEGs in JIN vs ZSH.

Supplement: Supplementary file 1 [file metabolites-13-00742-s001.zip › Figure S2.pdf]

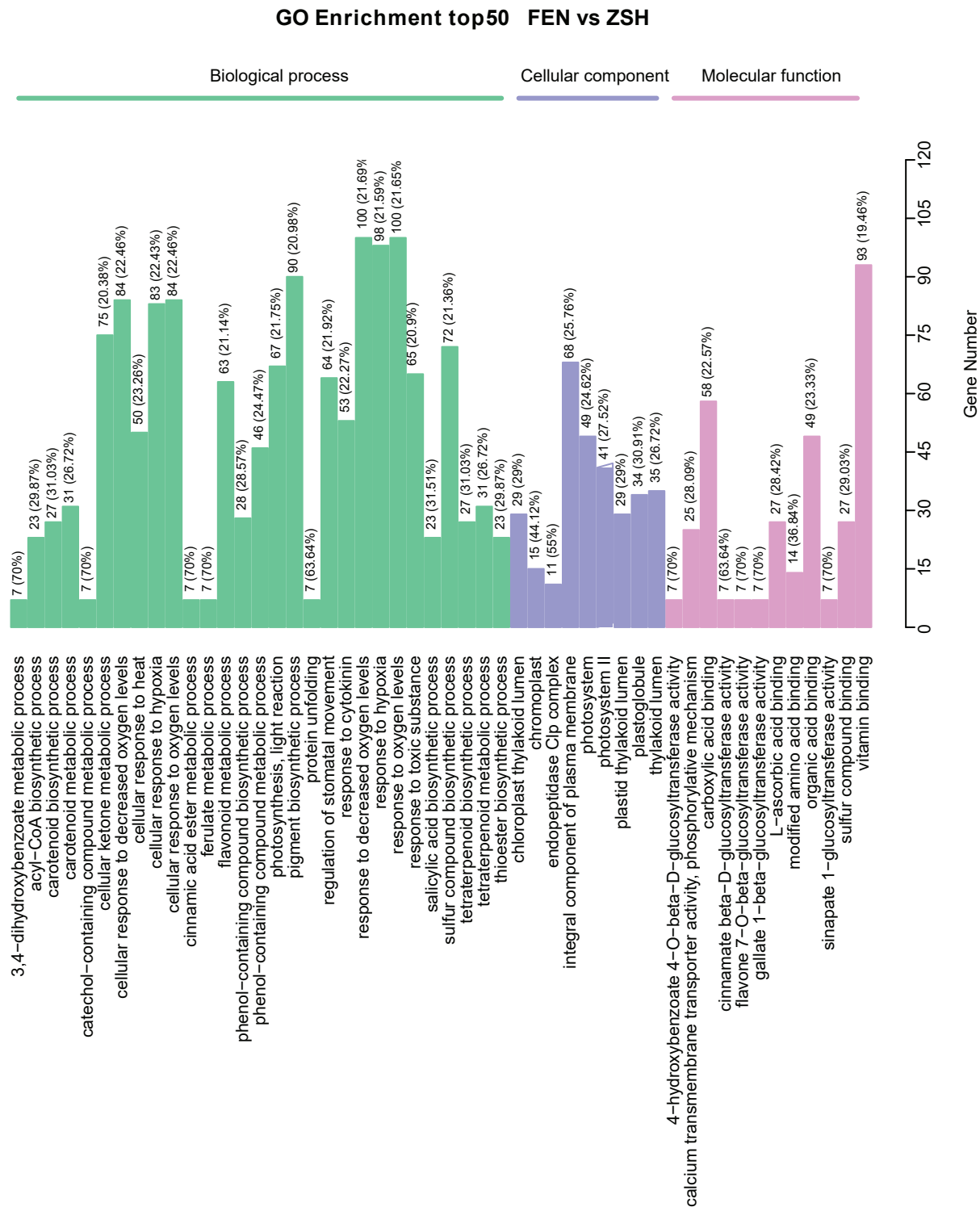

**Figure S3.** The GO enrichment of DEGs in FEN vs ZSH.

Supplement: Supplementary file 1 [file metabolites-13-00742-s001.zip › Figure S3.pdf]
